# Disparate properties of *Burkholderia multivorans* and *Pseudomonas aeruginosa* regarding outer membrane chemical permeabilization to the hydrophobic substances novobiocin and triclosan

**DOI:** 10.1371/journal.pone.0284855

**Published:** 2023-04-25

**Authors:** Sallie A. Ruskoski, Allison A. McDonald, Jeffrey J. Bleichner, Sheeba S. Aga, Kavya Boyina, Franklin R. Champlin

**Affiliations:** 1 Department of Biochemistry and Microbiology, Oklahoma State University Center for Health Sciences, Tulsa, OK, United States of America; 2 Department of Health Professions, Northeastern State University, Broken Arrow, OK, United States of America; 3 Department of Biotechnology, Tulsa Community College, Tulsa, OK, United States of America; University of Sadat City, EGYPT

## Abstract

*Burkholderia multivorans* causes opportunistic pulmonary infections and is intrinsically resistant to many antibacterial compounds including the hydrophobic biocide triclosan. Chemical permeabilization of the *Pseudomonas aeruginosa* outer membrane affects sensitization to hydrophobic substances. The purpose of the present study was to determine if *B*. *multivorans* is similarly susceptive suggesting that outer membrane impermeability properties underlie triclosan resistance. Antibiograms and conventional macrobroth dilution bioassays were employed to establish baseline susceptibility levels to hydrophobic antibacterial compounds. Outer membrane permeabilizers compound 48/80, polymyxin B, polymyxin B-nonapeptide, and ethylenediaminetetraacetic acid were used in attempts to sensitize disparate *B*. *multivorans* isolates to the hydrophobic agents novobiocin and triclosan, and to potentiate partitioning of the hydrophobic fluorescent probe 1-*N-*phenylnapthylamine (NPN). The lipophilic agent resistance profiles for all *B*. *multivorans* strains were essentially the same as that of *P*. *aeruginosa* except that they were resistant to polymyxin B. Moreover, they resisted sensitization to hydrophobic compounds and remained inaccessible to NPN when treated with outer membrane permeabilizers. These data support the notion that while both phylogenetically-related organisms exhibit general intrinsic resistance properties to hydrophobic substances, the outer membrane of *B*. *multivorans* either resists permeabilization by chemical modification or sensitization is mitigated by a supplemental mechanism not present in *P*. *aeruginosa*.

## Introduction

*Burkholderia multivorans* is a member of the *Burkholderia cepacia* complex (Bcc) and an opportunistic pulmonary pathogen in immunocompromised individuals, cystic fibrosis (CF), or chronic granulomatous disease (CGD) [1]. Patients infected with certain members of the Bcc are capable of developing cepacia-like syndrome; a progressive decline in lung function with potentially necrotizing pneumonia [2]. These organisms generally exhibit a high level of resistance to a large group of antibiotics [3], thereby potentiating their virulence. *B*. *multivorans* is presently the most common Bcc organism to be isolated from CF patients in the United States, as well as other countries such as Canada, Europe, and New Zealand [4]. The highly selective nature and general impermeability of the gram-negative outer membrane often underlies the refractory nature of these bacteria for hydrophobic molecules [5]. Solutes are able to passively diffuse into the periplasm by virtue of two primary mechanisms [6]. First, outer membrane porins allow for the selective passage of low molecular weight polar molecules such as nutrients. Second, the overall organization of the outer membrane, specifically the presence of lipopolysaccharide (LPS) in the outer leaflet coupled with porin discrimination for low molecular weight polar solutes, precludes diffusion of hydrophobic and bulky molecules in the absence of localized disruption or regions of phospholipid-LPS bilayer.

Outer membrane permeabilizers consist of compounds capable of disrupting the exclusionary properties of the gram-negative outer cell envelope [7]. In the case of hydrophobic molecules, they are thought to disrupt the asymmetric placement of intact LPS in the outer membrane outer leaflet, thereby creating regions of phospholipid bilayer which compromise its permeability barrier function [5, 8]. Compound 48/80 is a mixture of the polycationic polymer *p*-methoxyphenethylmethylamine originally used to promote histamine release in mast cells [9], but has subsequently been proven useful as an outer membrane permeabilizer [8, 10–12], by virtue of its ability to displaced Ca^2+^ from the surface of target cells [10]. The cationic compound polymyxin B disrupts exclusionary properties of the outer membrane [7, 13] by interacting with cation binding sites and partitioning into the outer membrane resulting in its localized disruption [14]. Polymyxin B-nonapeptide, which lacks the fatty acyl side chain of polymyxin B, also lacks its bactericidal potential while retaining its ability to selectively permeabilize the outer membrane [8, 11, 14]. Ethylenediaminetetraacetic acid (EDTA) is a divalent cation chelator that removes stabilizing Mg^2+^ molecules resulting in LPS release [15], thereby generating regions of phospholipid bilayer due to the compensatory filling of remaining outer leaflet voids with inner leaflet phospholipids to create a functional hydrophobic diffusion pathway into the periplasm [5].

Previous work revealed *P*. *aeruginosa* strains possessing either a highly refractory outer cell envelope, an atypically permeable outer cell envelope, or a multidrug efflux pump deficiency were sensitized to sub-minimal inhibitory concentrations of the hydrophobic antibacterial agents triclosan and novobiocin when treated with the mechanistically-disparate outer membrane permeabilizers compound 48/80, EDTA, and polymyxin B-nonapeptide [11]. These results strongly suggest that the outer membrane exclusionary properties for hydrophobic substances are responsible for the intrinsic resistance of *P*. *aeruginosa* to triclosan. Subsequent work revealed that sensitization by compound 48/80 was transitory due to the activity of triclosan-recognizing efflux pumps rather than obviation of the hydrophobic pathway [12]. Conversely, the marked susceptibility of *Pasteurella multocida* to hydrophobic substances and outer membrane permeability for the hydrophobic fluorescent probe 1-N-phenylnapthylamine (NPN) was shown to be due to the intrinsic inability of its outer membrane to exclude hydrophobic molecules in general [16], thereby strongly suggesting the presence of phospholipid bilayer regions in naturally-occurring cells. Contrarily, Moore and Hancock [17] were unable to chemically facilitate outer membrane permeability for NPN using either polymyxin B or EDTA in the bacterium *Pseudomonas* (now *Burkholderia*) *cepacia*.

A paucity of basic knowledge exists regarding the basic biology underlying the pathogenicity of *B*. *multivorans* due to its taxonomic novelty. Previous work has established that cell envelope membranes from disparate strains of *B*. *multivorans* are composed of similar phospholipid profiles, while their substituent fatty acyl groups differ quantitatively [18]. Moreover, expression of extracellular polysaccharide does not influence the degree to which they associate with nonpolar substances [19]. The present study was undertaken to determine if *B*. *multivorans* strains respond in a manner similar to *P*. *aeruginosa* to the sensitizing effects of chemically-dissimilar outer membrane permeabilizers with regard to the modification of their intrinsic resistance to the hydrophobic antibacterial agents novobiocin and triclosan.

## Materials and methods

### Bacterial strains and maintenance

*P*. *aeruginosa* PAO1, *P*. *multocida* ATCC 11039 (American Type Culture Collection, Manassas, VA), and CF clinical type strain *B*. *multivorans* ATCC BAA-247 (American Type Culture Collection) are kept as reference organisms in this laboratory. *B*. *multivorans* environmental strain ATCC 17616 and clinical CGD isolate CGD2 were provided by Dr. Adrian Zelazny (NIH-NIAID, Bethesda, MD). All cultures were maintained under cryoprotective conditions at -80˚C [20] to provide inocula for working cultures which were cultivated on Difco Mueller Hinton Agar (MHA; Beckton Dickinson Co., Sparks, MD). Starter cultures were prepared by inoculating Difco Mueller Hinton Broth (MHB; Beckton Dickinson Co.) with cells from working cultures as described previously [20].

### Disk agar diffusion bioassay

Antibiograms of hydrophobic antibacterial agents for each organism were obtained using a standardized disk agar diffusion bioassay as described previously [21] using MHA plates and BL Sensi-Disc (Beckton Dickinson Co.) susceptibility test disks. Triclosan-containing disks were prepared by aseptically impregnating sterile paper blanks (6.0-mm diameter; Becton Dickinson Co.) with 0.2 μg of triclosan (Irgasan DP 300; Ciba Specialty Chemicals Corp., High Point, NC) each in 95% ethanol solution and dried overnight under sterile flowing air.

### Macrobroth dilution bioassay

Minimal inhibitory concentrations (MICs) were determined using a modified conventional macrobroth two-fold dilution bioassay [11, 20] with MHB as diluent. MHB stock solutions of novobiocin sodium (Sigma-Aldrich Co., St Louis, MO), rifamycin SV (Sigma-Aldrich Co.), and polymyxin B sulfate (Sigma-Aldrich Co.) were prepared to desired concentrations, filter sterilized (0.22 μm Fisherbrand Syringe Filter; Thermo Fisher Scientific Inc., Pittsburgh, PA), and stored at 4°C until needed. A stock solution (1,280 μg/mL) of triclosan was prepared in 95% ethanol and diluted to a concentration of 128 μg/mL in sterile MHB in a manner such that the final ethanol concentration never exceeded 0.4%. Minimal bactericidal concentrations (MBCs) were determined by inoculating MHA plates with 100 μl from concentrations greater than those contained in the MIC endpoint cultures and incubating for 24 h at 37°C before visually assessing colonial growth.

### Outer membrane permeabilization bioassay

Synergistic growth inhibition by combinations of outer membrane permeabilizers and either novobiocin or triclosan was assessed using turbidimetric measurements of batch cultural growth kinetics as previously employed in this laboratory [11–13, 16]. Triclosan was dissolved in 95% ethanol to a concentration of 500 μg/mL such that the addition of small volumes to MHB test cultures yielded desired final concentrations with the ethanol concentration never exceeding 0.4%. Stock solutions of the outer membrane permeabilizers compound 48/80 (Sigma-Aldrich Co.), polymyxin B-nonapeptide (Sigma-Aldrich Co.), polymyxin B sulfate (Sigma-Aldrich Co.), and EDTA (Sigma-Aldrich Co.) were prepared to desired concentrations in MHB and filter sterilized (0.22 μm Fisherbrand Syringe Filter). Overnight MHB starter and MHB experimental cultures containing either novobiocin or triclosan with indicated outer membrane permeabilizers were prepared as before [11] and growth was assessed by monitoring optical density at 620 nm (OD_620_) with the aid of a Spectronic 20D+ optical spectrophotometer (Thermo Fischer Scientific Inc.) at 0.5 h intervals for 6 h. Outer membrane permeabilization was determined on the basis of observable sensitization of otherwise refractory organisms to less than MIC levels of the hydrophobic antibacterial agents novobiocin and triclosan.

### NPN uptake assay

Potentiation of the accessibility of hydrophobic regions of the outer membrane to the hydrophobic fluorescent probe NPN (Sigma-Aldrich Co.) by outer membrane permeabilizers was assessed using the method of Helander and Matilla-Sandholm (2000) [22] as modified for use in this laboratory [16]. Late exponential-phase cells cultured in the presence of various outer membrane permeabilizers (described above) were harvested by centrifugation for 20 min at 10,000 x g and 4°C (Sorvall Legend XTR Centrifuge; Thermo Fisher Scientific Inc.), and suspended to an OD_620_ of 0.5 in 0.5 mM HEPES buffer (pH 7.2; Sigma-Aldrich Co.). NPN and experimental treatments were prepared as described previously [16] in 96-well microtiter plates (Costar 96-well black, clear bottom microtiter plates; Corning Inc., Lowell, MA). Fluorescence was measured using a Synergy 2 Multi-Detection Microplate Reader (BioTek Instruments Inc., Winooski, VT) with excitation and emission wavelengths of 340 nm and 415 nm, respectively. Relative fluorescence was calculated on the basis of the ratio of differences between experimental treatment value and organism control value to the differences between the NPN control value and the HEPES control value. Statistically significant differences between treatment group means were determined using a one-way ANOVA with Tukey’s post-hoc pairwise comparisons.

## Results and discussion

### Susceptibility to hydrophobic antibacterial agents

A standardized disk agar diffusion bioassay was employed to determine general susceptibility levels of the *B*. *multivorans* strains under study to hydrophobic and lipophilic antibacterial molecules (Table 1). The refractory organism *P*. *aeruginosa* PAO1 was resistant to all but the lipopeptide polymyxin B, while the negative control organism *P*. *multocida* ATCC 11039 was susceptible to all of the antibacterial agents examined except for oxacillin. This is consistent with our previous demonstrations that the atypically permeable outer membrane of *P*. *multocida* renders it markedly susceptible to the hydrophobic biocide triclosan [16, 21], while the exclusionary properties of the *P*. *aeruginosa* outer cell envelope are largely responsible for its high level of intrinsic resistance to hydrophobic substances in general [11–13]. The antibiograms for the *B*. *multivorans* strains were essentially the same as for *P*. *aeruginosa* with the exception of polymyxin B. The resistance to polymyxin B observed in the *B*. *multivorans* strains is consistent with a previous report that this phenotypic trait can be employed to differentiate them from *P*. *aeruginosa* [23].

**Table 1:** Susceptibility to outer membrane impermeant antibacterial agents and polymyxin B.

		Inhibition zone diameter (mm) ± SD^a^							MIC (μg*/*ml)^c^
Organism	Control	TCS	NOV	RIF	CLI	VAN	OXA	PMB	PMB
*P*. *aeruginosa*									<0.125
PA01	0	0^c^	1.6±0.3	0	0	0	0	9.8±1.0	
*P*. *multocida*									nd
ATCC 11039	0	16.9±5.7	34.7±2.9	23.6±1.7	6.0±3.2	34.7±2.9	1.4±1.4	12.2±1.0	
*B*. *multivorans*									
ATCC BAA-247	0	0	4.4±1.2	0.8±4.9	0	0	0	0	>1024.0
ATCC 17616	0	0	6.4±1.6	5.0±1.1	0	0	0	2.8±0.2	1024.0
CGD2	0	0	4.8±0.9	2.2±0.5	0	0.9±0.1	0	0	>1024.0

^a^ Diameter of growth inhibition zone minus disk diameter (6 mm). Each value represents the mean of three independent determinations ± SD using a standardized disk agar diffusion bioassay. Abbreviations (potencies): TCS, triclosan (0.2 μg); NOV, novobiocin (5.0 μg); RIF, rifampin (5.0 μg); CLI, clindamycin (2.0 μg); VAN, vancomycin (30 μg); OXA, oxacillin (1.0 μg); PMB, polymyxin B (30 μg).

^b^ Ethanol (95%) was used to facilitate triclosan solubilization, therefore control disks were prepared by impregnating with solvent alone and allowing to air dry prior to plate application.

^c^Each value was obtained from three independent two-fold serial dilutions using a conventional macrobroth dilution bioassay.

These data are consistent with a previous report [19] using the macrobroth dilution bioassay method to generate MIC values. The refractory organism *P*. *aeruginosa* was inordinately resistant to the hydrophobic, mechanistically-disparate molecules novobiocin, triclosan, and rifamycin SV, while *P*. *multocida* was susceptible to extremely low levels of all three. The three *B*. *multivorans* strains were also resistant to all three compounds, although much less so for novobiocin when compared to *P*. *aeruginosa*. They can be seen in the present study to be highly resistant to polymyxin B in contrast with *P*. *aeruginosa* with MIC values of 1024 μg/mL and greater (Table 1). Because the hydrophobic and lipophilic antibacterial agents examined possess mechanistically-disparate cell targets, the intrinsic resistance of *B*. *multivorans* may likely be attributed to the general impermeability of its otherwise ultrastructurally-typical [24] outer membrane.

### Sensitization to hydrophobic antibacterial agents

**Fig 1:**
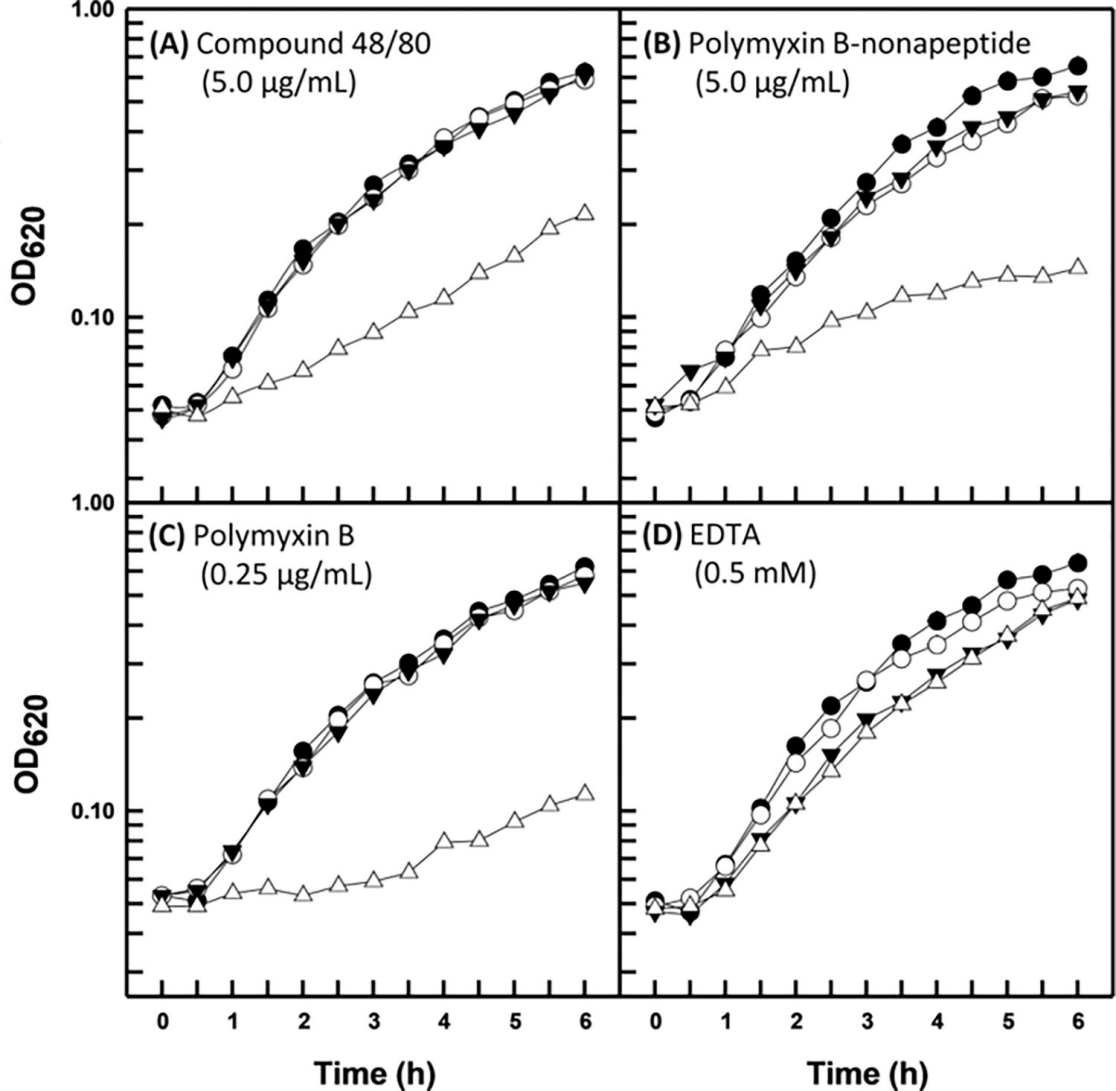
Growth kinetics of *P*. *aeruginosa* PAO1 in the presence of novobiocin (10.0 μg/mL) and the indicated outer membrane permeabilizer. Each value represents the mean of at least three independent determinations. Symbols: (●) control, (○) novobiocin, (▼) permeabilizer, (△) novobiocin plus permeabilizer.

**Fig 2:**
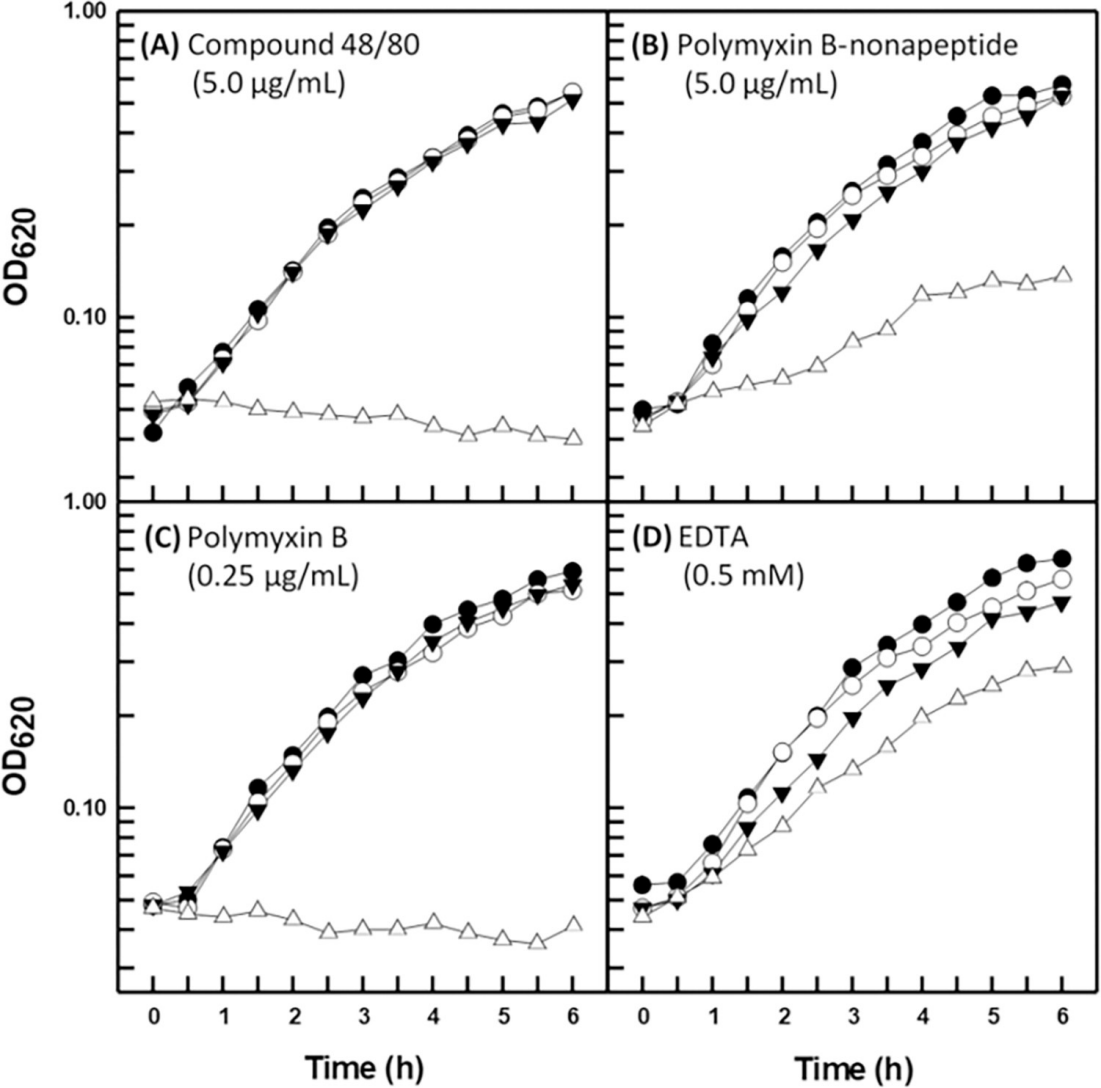
Growth kinetics of *P*. *aeruginosa* PAO1 in the presence of triclosan (2.0 μg/mL) and the indicated outer membrane permeabilizer. Each value represents the mean of at least three independent determinations. Symbols: (●) control, (○) triclosan, (▼) permeabilizer, (△) triclosan plus permeabilizer.

In order to determine if the outer cell envelope of *B*. *multivorans* functions to retard the diffusion of the hydrophobic antibacterial agents novobiocin and triclosan into the periplasm, four compounds known to permeabilize gram-negative outer membranes to otherwise excluded antibacterial compounds [7, 11] were examined for growth-inhibitory synergism. The structural and functional disparities of the permeabilizers mitigated potential ambiguous results given the possibility that any one compound could potentiate deleterious secondary effects resulting in growth inhibition. As expected, outer membrane permeabilizers compound 48/80, polymyxin B-nonapeptide, and polymyxin B exhibited synergistic relationships with novobiocin (Fig 1A–1C) and triclosan (Fig 2A–2C) for the susceptive control organism *P*. *aeruginosa* PA01 as demonstrated by turbidimetric measurements of batch cultural growth. However, EDTA proved slightly growth inhibitory and failed to sensitize the organism to novobiocin (Fig 1D), while acting only somewhat synergistically with triclosan (Fig 2D). This result is consistent with that reported previously by Champlin *et al*. [11] who found that EDTA at a two-fold greater concentration moderately inhibited the growth of strain PAO1, while also moderately sensitizing it to triclosan.

**Fig 3:**
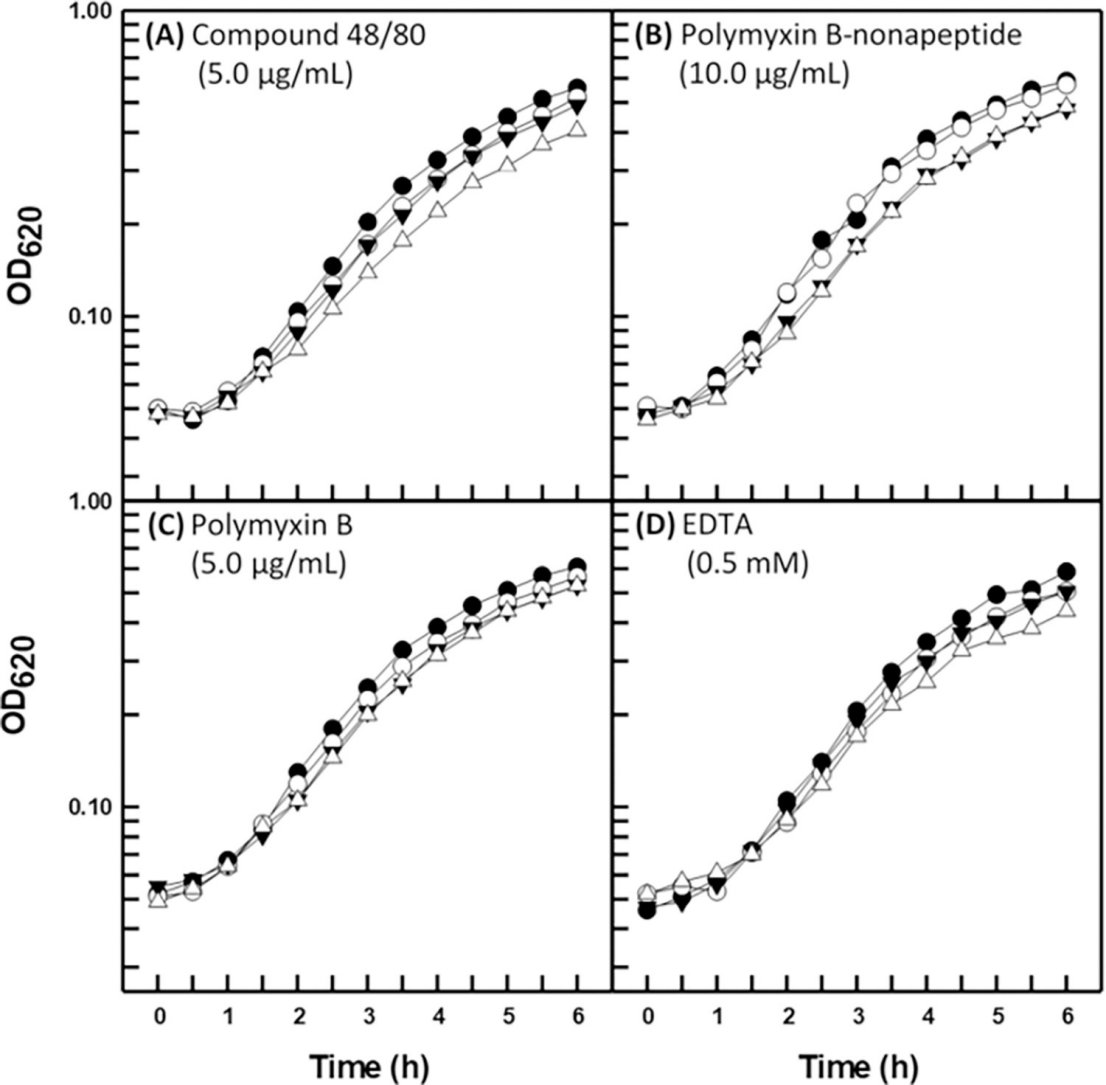
Growth kinetics of *B*. *multivorans* ATCC BAA-247 (type strain) in the presence of novobiocin (1.0 μg/mL) and the indicated outer membrane permeabilizer. Each value represents the mean of at least three independent determinations. Symbols: (●) control, (○) novobiocin, (▼) permeabilizer, (△) novobiocin plus permeabilizer.

**Fig 4:**
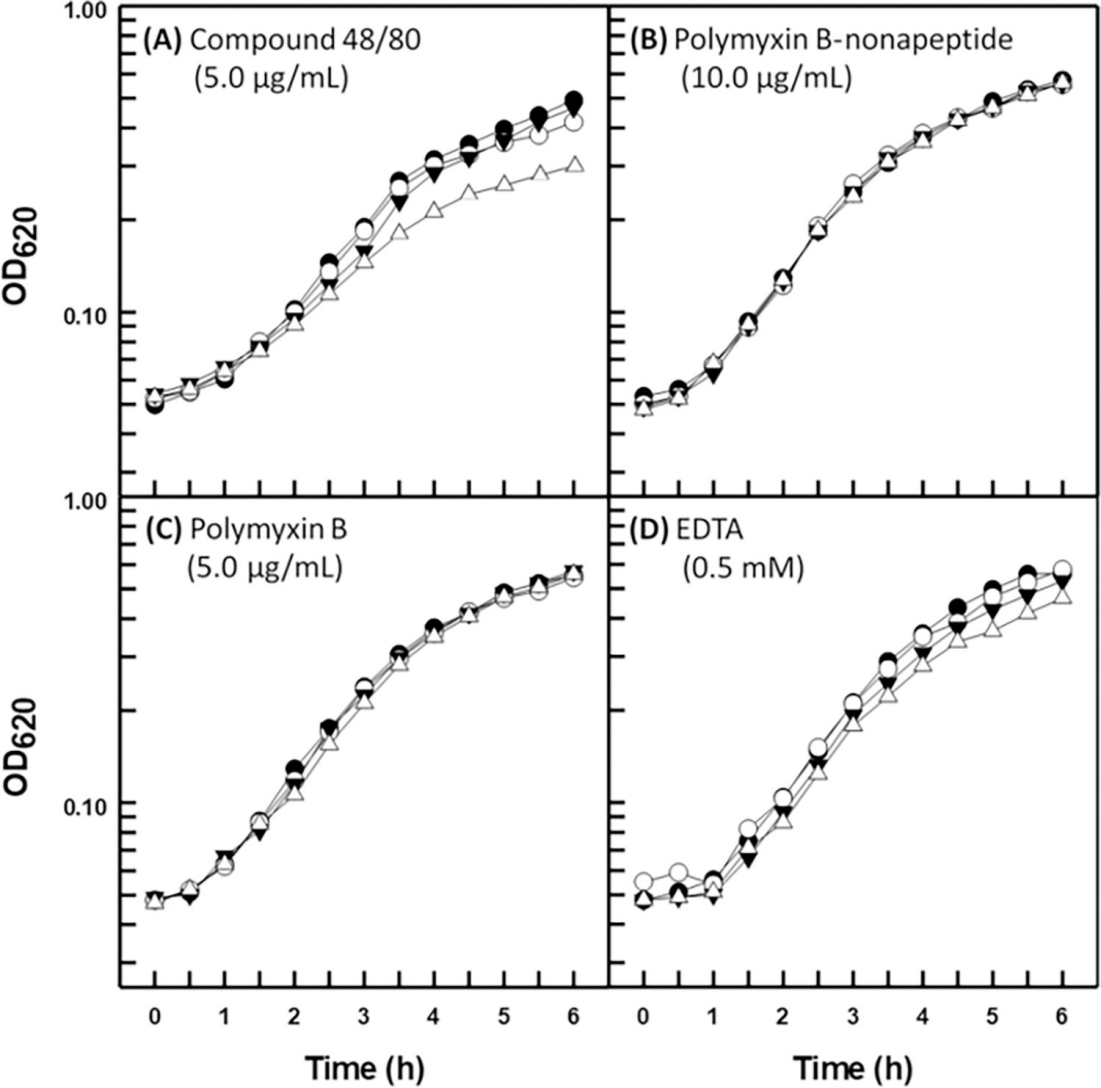
Growth kinetics of *B*. *multivorans* ATCC BAA-247 (type strain) in the presence of triclosan (1.0 μg/mL) and the indicated outer membrane permeabilizer. Each value represents the mean of at least three independent determinations. Symbols: (●) control, (○) triclosan, (▼) permeabilizer, (△) triclosan plus permeabilizer.

The three disparate strains of *B*. *multivorans* proved refractory to the permeabilization properties of all four outer membrane permeabilizers for both novobiocin and triclosan. Representative data are shown for type strain BAA-247 in Figs 3 and 4, respectively (data in S1–S4 Figs for strains ATCC 17616 and CGD2), and can be seen to be in direct contrast with those obtained for *P*. *aeruginosa* (Figs 1 and 2). One exception exists in that compound 48/80 appeared to slightly sensitize strain BAA-247 to novobiocin and triclosan (Figs 3A and 4A) at the same concentration that clearly permeabilized the *P*. *aeruginosa* PAO1 outer membrane to both hydrophobic probes (Figs 1A and 2A). It is interesting to note that strains ATCC 17616 and CGD2 appeared slightly susceptible to EDTA (data in S1–S4 Figs) in much the same way as *P*. *aeruginosa* PAO1 (Fig 1D) [11]. Polymyxin B treatments were employed at greater concentrations (Figs 3C and 4C) for the B*urkholderia* strains because they are markedly more resistant to polymyxin B than is *P*.*aeruginosa* (Table 1). Polymyxin B-nonapeptide, a hydrolytic derivative of polymyxin B, was also used at a greater concentration (Figs 3B and 4B) for the polymyxin B-resistant *B*. *multivorans*. Both chemicals possess the same outer membrane binding mechanism with polymyxin B-nonapeptide lacking the bactericidal potential of its parent compound [8].

The four outer membrane permeabilizers employed here represent both different chemical structures and mechanisms of action. Moreover, the three *B*. *multivorans* strains employed for this study represent independent isolates of CF, environmental, and CGD origin. These data then suggest that the outer cell envelope of *B*. *multivorans* generally resists chemical perturbation of its exclusionary properties for hydrophobic antibacterial agents by virtue of a general mechanism which is conserved among diverse strains and is absent in the phylogenetically closely-related organism *P*. *aeruginosa*.

### Potentiation of NPN uptake

**Fig 5:**
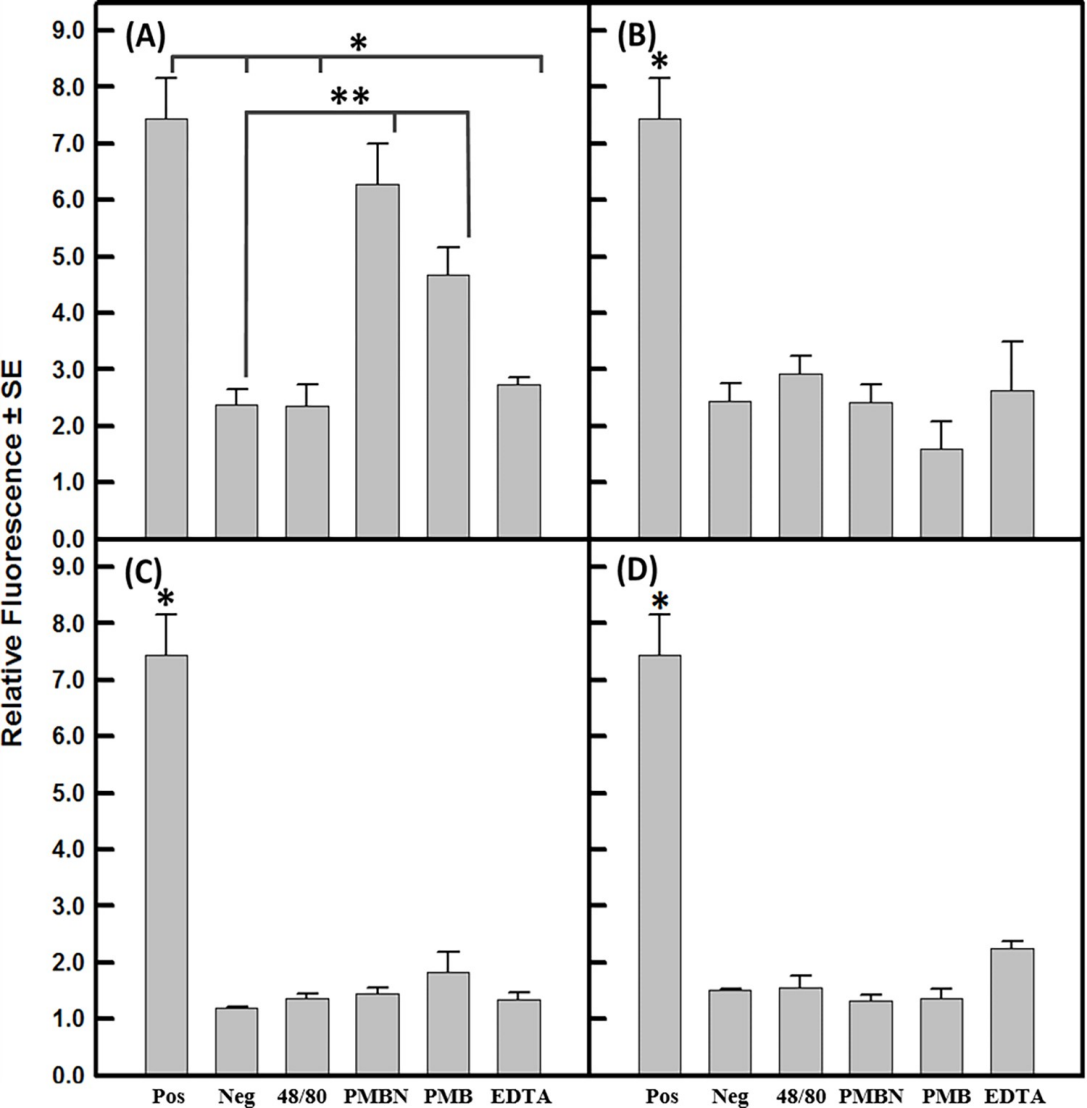
Potentiation of NPN uptake by outer membrane permeabilization of test organisms. Each value represents the mean of at least three independent determinations plus standard error. (A) *P*. *aeruginosa* PAO1, (B) *B*. *multivorans* BAA-247, (C) *B*. *multivorans* ATCC 17616, and (D) *B*. *multivorans* CGD2. Abbreviations: Pos, *P*. *multocida* ATCC 11039 (positive control); Neg, cells lacking permeabilizer (negative control); 48/80, compound 48/80; PMBN, polymyxin B nonapeptide; PMB, polymyxin B; and EDTA, ethylenediaminetetracetic acid. Comparisons represented by * indicate significant differences (P < 0.05) between positive control and treatment groups, while ** indicate significant differences (P < 0.05) between negative control and treatment groups. Statistically significant differences between treatment group means was determined using a one-way ANOVA with Tukey’s post-hoc pairwise comparisons.

The apparent inability of the *B*. *multivorans* outer membrane to undergo chemical permeabilization to nonpolar antibacterial compounds was further investigated using the hydrophobic probe NPN. The degree of fluorescence emitted by NPN is a function of the degree to which it is able to associate with and partition into hydrophobic domains [22] and is therefore an indicator of the presence of localized regions of cell surface hydrophobicity [5, 25]. *P*. *multocida* ATCC 11039 was employed as a positive control due to the marked permeability of its outer cell envelope for hydrophobic substances and the degree to which NPN is able to partition into its outer cell envelope in the absence of outer membrane permeabilization [16]. The refractory organism *P*. *aeruginosa* PAO1 was rendered permeable to NPN at levels statistically similar (P > 0.05) to that of the positive control organism *P*. *multocida* when treated with either of the outer membrane permeabilizers polymyxin B-nonapeptide or polymyxin B (Fig 5A). These results are consistent with those observed in Figs 1B, 1C, 2B and 2C where they can be seen to also be synergistic with both novobiocin and triclosan. However, neither compound 48/80 nor EDTA elicited a statistically significant (P > 0.05) increase in NPN permeabilization when compared with the untreated negative control *P*. *aeruginosa* PAO1. The degree of compound 48/80 and EDTA-induced permeabilization to the *P*. *aeruginosa* outer membrane rendered it permeable to both novobiocin and triclosan (Figs 1A and 2A, respectively). EDTA similarly rendered the organism susceptible to triclosan (Fig 2D). Yet the modifications elicited by neither permeabilizer were apparently sufficient to allow for the association of NPN with the cell surface (Fig 5A).

None of the four outer membrane permeabilizers were able to significantly (P > 0.05) sensitize *B*. *multivorans* strains BAA-247, ATCC 17616, or CGD2 to NPN when compared to their negative controls (Fig 5B–5D). Furthermore, all *B*. *multivorans* treatment groups associated with NPN at levels significantly (P < 0.05) less than the positive control *P*. *multocida* ATCC 11039. These results further support the notion that the outer membrane of *B*. *multivorans* resists chemical permeabilization for hydrophobic substances because NPN is not dependent upon a mechanistic cell target and fluoresces only when associated with hydrophobic cell surface regions suggestive of the hydrophobic pathway of outer membrane diffusion.

Because three disparate *B*. *multivorans* strains proved uniformly resistant to diverse hydrophobic agents representing multiple different mechanisms of action in a manner essentially identical to that of *P*. *aeruginosa* (Table 1), it can be surmised that they too possess outer membranes generally impermeable to hydrophobic substances. However, in contrast with the propensity of *P*. *aeruginosa* to undergo sensitization to both novobiocin and triclosan by virtue of chemical disruption of its outer membrane impermeability properties for hydrophobic compounds [11, 12, 26], the *B*. *multivorans* outer cell envelope remained refractory to hydrophobic substances when treated with similar concentrations of four disparate compounds capable of obviating the exclusionary properties of other bacteria [7, 11]. *Pseudomonas* (now *Burkholderia*) *cepacia* has similarly been shown to resist permeabilization to the hydrophobic fluorescent probe NPN with either polymyxin B or EDTA [17], thereby suggesting genus-level differences in outer cell envelope physiology between these otherwise phylogenetically closely-related organisms. The disparate CF, environmental, and CGD origins of the strains examined suggest *B*. *multivorans* generally possesses an extremely impermeable outer membrane and is consistent with its opportunistic nosocomial properties. Finally, *P*. *aeruginosa* was shown to be susceptible to polymyxin B and susceptive to its permeabilization effects while three disparate polymyxin B-resistant *B*. *multivorans* strains were resistant to both. This relationship may likely be attributed to phenotypic differences in the availability or affinity of LPS binding sites for cationic lipopeptides or specific porinis in the outer membrane [27] in these closely-related opportunistic bacterial pathogens.

## Conclusion

This work supports the notion that both of the phylogenetically related bacteria *P*. *aeruginosa* and *B*. *multivorans* possess outer cell envelopes which are intrinsically refractory for hydrophobic substances. *B*. *multivorans* is non-susceptive to outer membrane permeabilization by chemical modification or such sensitization is mitigated by a supplemental intrinsic resistance mechanism not present in *P*. *aeruginosa*.

## Supporting information

S1 Fig Growth kinetics of *B*. *multivorans* ATCC 17616 in the presence of novobiocin (1.0 μg/ml) and the indicated outer membrane permeabilizer. Each value represents the mean of at least three independent determinations. Symbols: (●) control, (▼) novobiocin, (■) permeabilizer, (♦) novobiocin plus permeabilizer.
(TIF)

S2 Fig Growth kinetics of *B*. *multivorans* ATCC 17616 in the presence of triclosan (1.0 μg/ml) and the indicated outer membrane permeabilizer. Each value represents the mean of at least three independent determinations. Symbols: (●) control, (▼) triclosan, (■) permeabilizer, (♦) triclosan plus permeabilizer.
(TIF)

S3 Fig Growth kinetics of *B*. *multivorans* CGD2 in the presence of novobiocin (1.0 μg/ml) and the indicated outer membrane permeabilizer. Each value represents the mean of at least three independent determinations. Symbols: (●) control, (▼) novobiocin, (■) permeabilizer, (♦) novobiocin plus permeabilizer.
(TIF)

S4 Fig Growth kinetics of *B*. *multivorans* CGD2 in the presence of triclosan (1.0 μg/ml) and the indicated outer membrane permeabililzer. Each value represents the mean of at least three independent determinations. Symbols: (●) control, (▼) triclosan, (■) permeabilizer, (♦) novobiocin plus permeabilizer.
(TIF)
