# Supplementary figures and images for: Disparate properties of Burkholderia multivorans and Pseudomonas aeruginosa regarding outer membrane chemical permeabilization to the hydrophobic substances novobiocin and triclosan
Source: PLoS One. 2023 Apr 25;18(4):e0284855. doi: 10.1371/journal.pone.0284855 (PMC10128999; doi:10.1371/journal.pone.0284855)

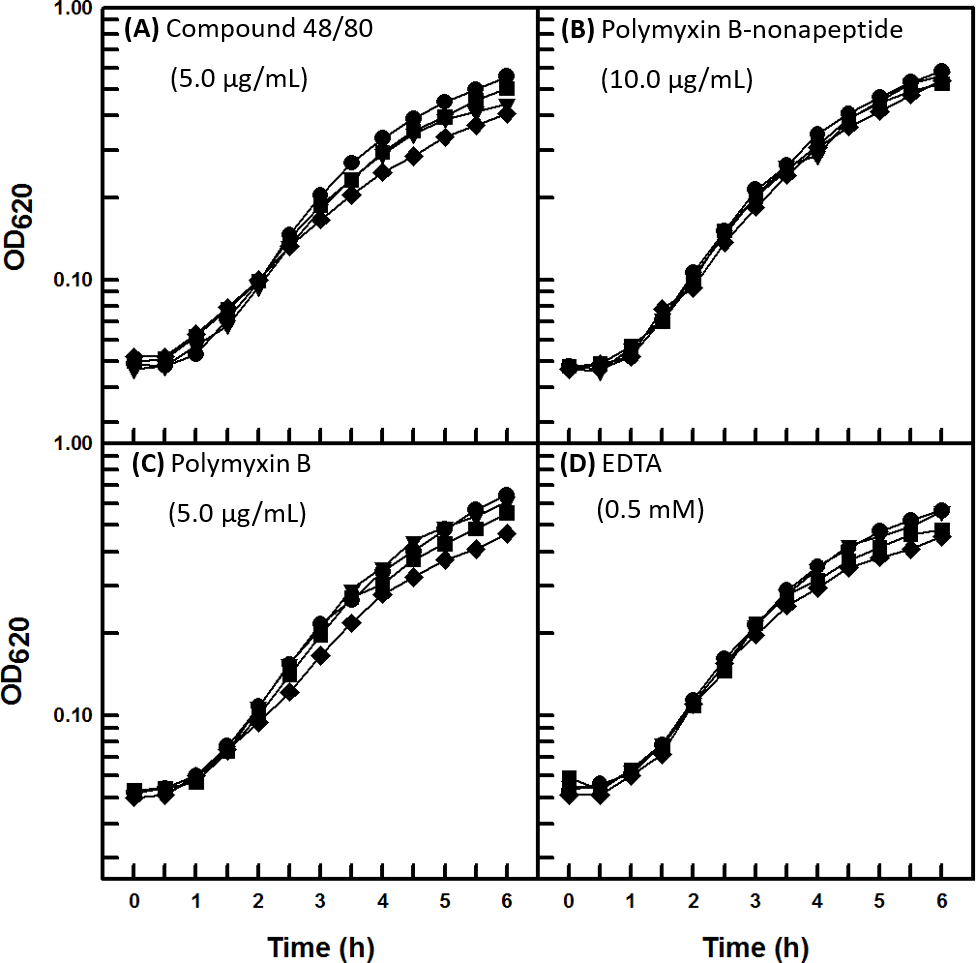

Supplement: S1 Fig — Each value represents the mean of at least three independent determinations. Symbols: (●) control, (▼) novobiocin, (■) permeabilizer, (♦) novobiocin plus permeabilizer. (TIF) [file pone.0284855.s001.tif]

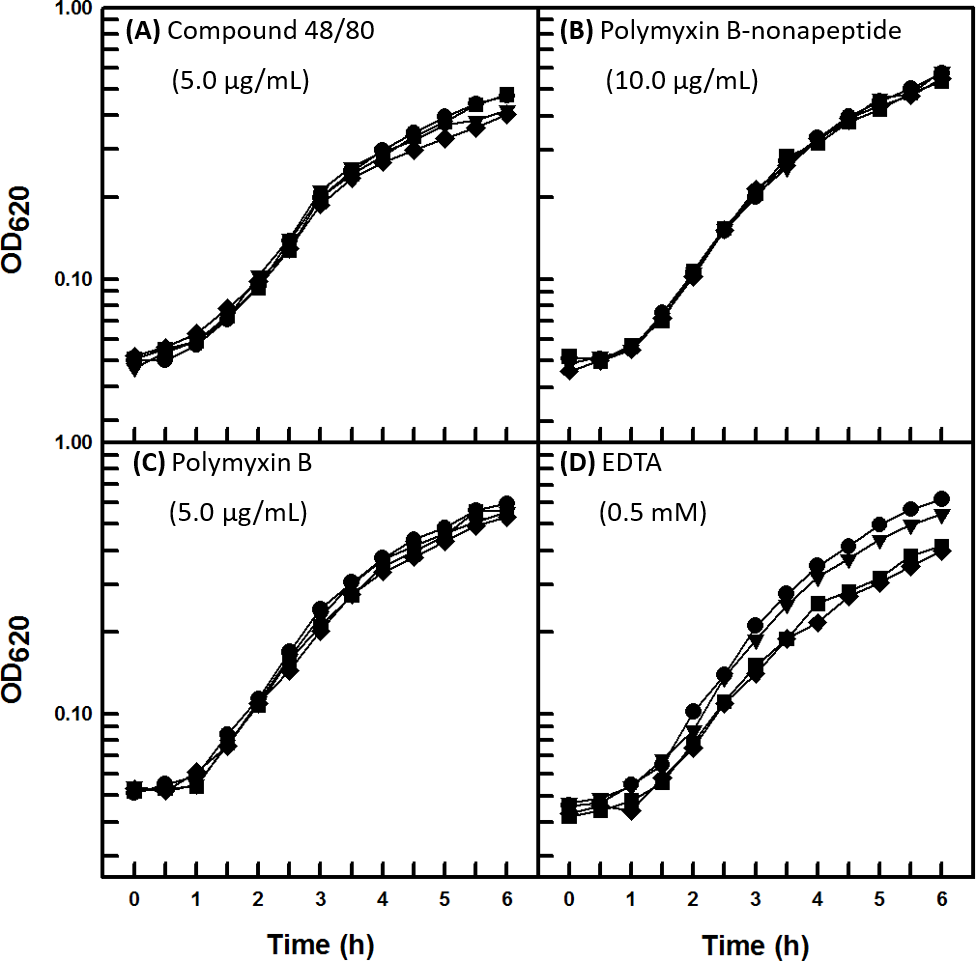

Supplement: S2 Fig — Each value represents the mean of at least three independent determinations. Symbols: (●) control, (▼) triclosan, (■) permeabilizer, (♦) triclosan plus permeabilizer. (TIF) [file pone.0284855.s002.tif]

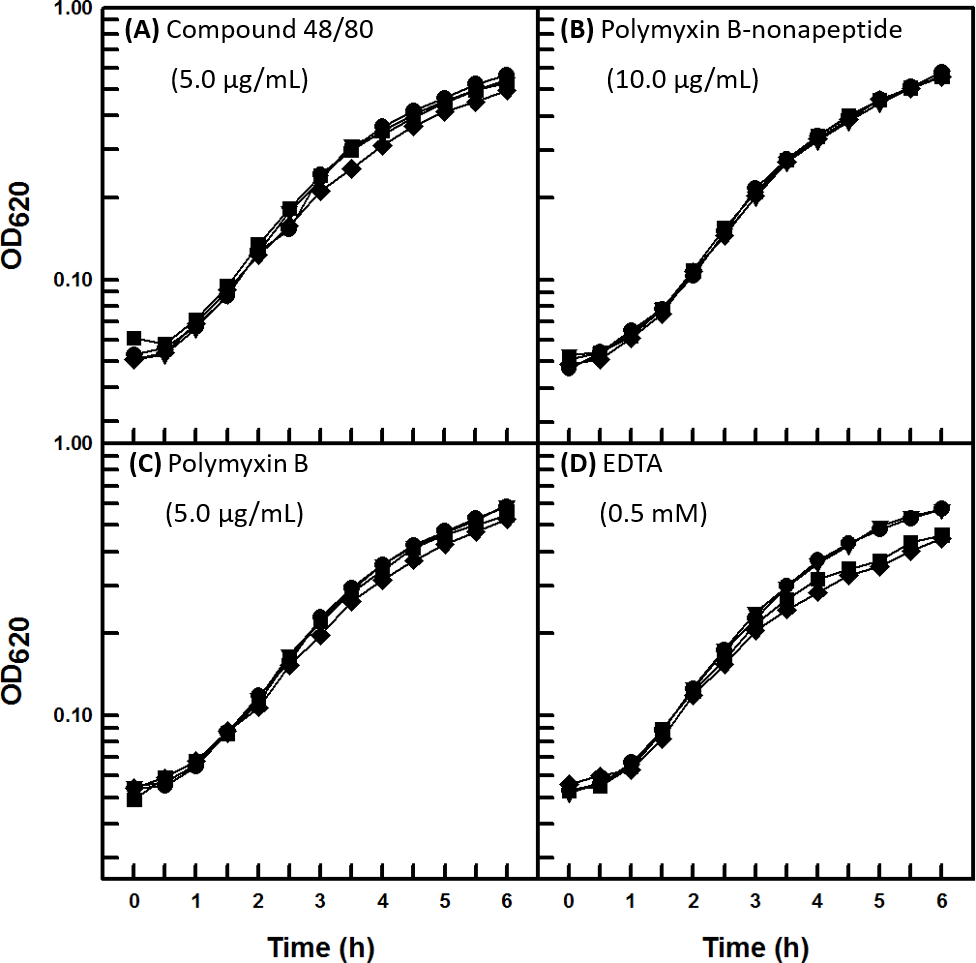

Supplement: S3 Fig — Each value represents the mean of at least three independent determinations. Symbols: (●) control, (▼) novobiocin, (■) permeabilizer, (♦) novobiocin plus permeabilizer. (TIF) [file pone.0284855.s003.tif]

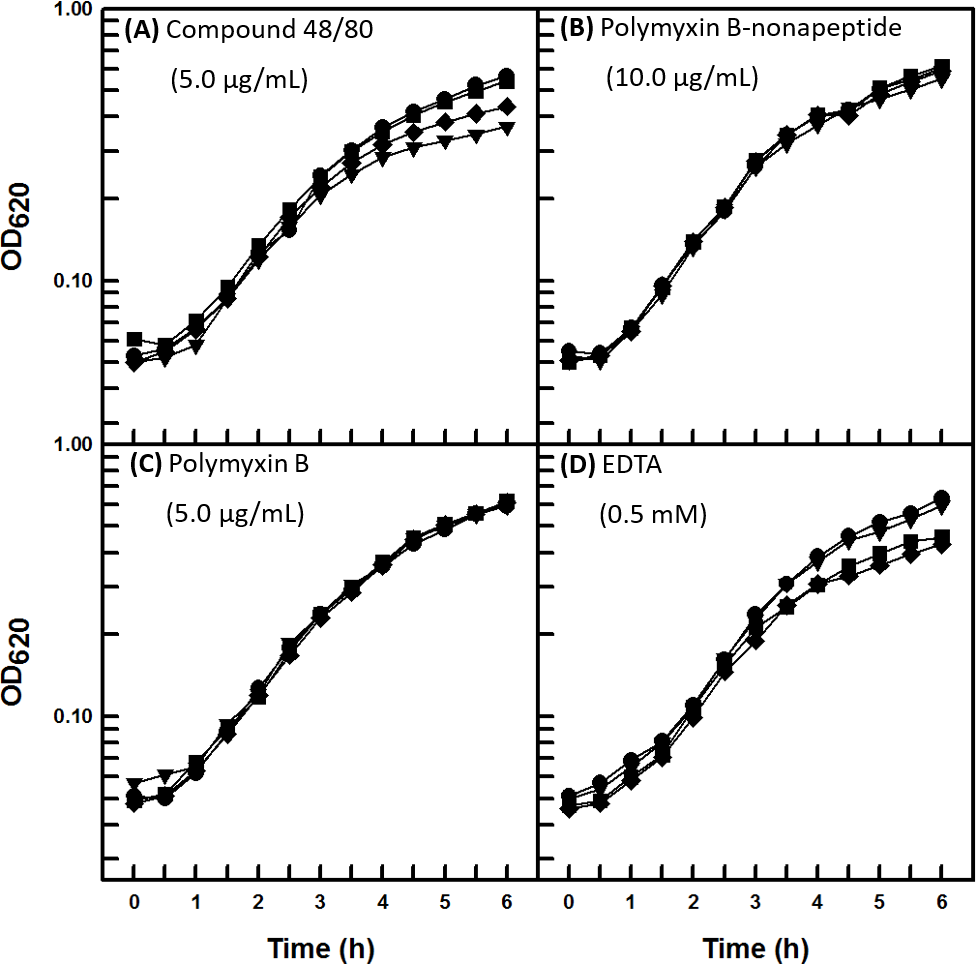

Supplement: S4 Fig — Each value represents the mean of at least three independent determinations. Symbols: (●) control, (▼) triclosan, (■) permeabilizer, (♦) novobiocin plus permeabilizer. (TIF) [file pone.0284855.s004.tif]
